# Non-Local Vectorial Internal Variables and Generalized Guyer-Krumhansl Evolution Equations for the Heat Flux

**DOI:** 10.3390/e25091259

**Published:** 2023-08-24

**Authors:** Liliana Restuccia, David Jou

**Affiliations:** 1 Department of Mathematical and Computer Sciences, Physical Sciences and Earth Sciences, University of Messina, Viale F. Stagno d’Alcontres, Salita Sperone 31, 98166 Messina, Italy; 2 Grup de Fisíca Estadística, Universitat Autònoma de Barcelona, 08193 Bellaterra, Spain; david.jou.mirabent@gmail.com; 3 Institut d’Estudis Catalans, Carme 47, 08001 Barcelona, Spain

**Keywords:** continuum thermodynamics, heat transport, classical irreversible thermodynamics, internal variables, phonon hydrodynamics

## Abstract

In this paper, we ask ourselves how non-local effects affect the description of thermodynamic systems with internal variables. Usually, one assumes that the internal variables are local, but that their evolution equations are non-local, i.e., for instance, that their evolution equations contain non-local differential terms (gradients, Laplacians) or integral terms with memory kernels. In contrast to this typical situation, which has led to substantial progress in several fields, we ask ourselves whether in some cases it would be convenient to start from non-local internal variables with non-local evolution equations. We examine this point by considering three main lengths: the observation scale *R* defining the elementary volumes used in the description of the system, the mean free path *l* of the microscopic elements of the fluid (particles, phonons, photons, and molecules), and the overall characteristic size *L* of the global system. We illustrate these ideas by considering three-dimensional rigid heat conductors within the regime of phonon hydrodynamics in the presence of thermal vortices. In particular, we obtain a generalization of the Guyer–Krumhansl equation, which may be of interest for heat transport in nanosystems or in systems with small-scale inhomogeneities.

## 1. Introduction

Non-local effects in heat transport (and in other transport phenomena) may arise for five physical reasons: (1) a long mean-free path of the heat carriers (phonons); (2) strongly inhomogeneous systems (such as foams or rocks); (3) long defects (such as dislocation lines) or systems of long lines (longitudinally conducting polymers); (4) artificially structured media; and (5) long correlations (as in superfluids or in critical states). The non-local effects may affect: (i) the definition of the local variable; (ii) the fluxes appearing in the equations; (iii) the form of the evolution equations; and (iv) the influence of the boundaries of the system.

In several cases, one must specify the size of the elementary volume cells used for the basic description of the system, for example, size *R*. There are two other reference lengths: the mean free path *l* and the characteristic volume size *L*. The mean free path depends on temperature, *L* is given by the size of the system (for instance, it could be the radius of a cylindrical channel), and *R* will be decided by the researcher in terms of the physical features of the system and of the observational range of interest or the range of the available observational instruments. If *l* is smaller than *R* and *L* or if *R* is much smaller than *l* and *l* much smaller than *L*, then the non-local effects will be negligible and the local equilibrium situation will be valid (the local equilibrium situation requires that the observation scale be larger than *l*, such that in any elementary volume there is a high number of collisions of the heat carriers leading the system to the local equilibrium state). The situation when *R* is much smaller than *l* but *l* is comparable to *L* has been studied on several occasions—for instance, in phonon hydrodynamics. Here, we aim to emphasize the conceptual complexity of the general description and suggest some points of reflection. Our motivations arise from our interest in thermal vortices but go beyond this concrete problem.

For instance, the presence of non-local effects in generalized equations for heat transport is of special interest in systems in which *l* is comparable to (or bigger than) *L*, i.e., for Knudsen number $\frac{l}{L}\geq 1$. This may be when the system is small enough that its size becomes comparable to the mean free path, as in nanosystems, or when the mean free path is sufficiently long that it becomes comparable to the size of the system, as is the case with phonons at low temperature, or in rarefied systems, as in aerodynamics at low environmental pressure. Some of the most-used phenomenological generalized heat transport equations in nanosystems are variations of the so-called Guyer–Krumhansl equation [1,2], which is a generalization of the relaxational Maxwell–Cattaneo–Vernotte equation [3,4,5], plus a Laplacian term in the heat flux $J^{\left(q\right)}$, times a coefficient proportional to the square of the mean free-path. Such an equation, plus some suitable boundary conditions on the heat flux at the walls of the system, giving, for instance, a slip heat flow along the walls, are the basis of phenomenological versions of phonon hydrodynamics [6,7,8,9,10,11,12,13,14,15,16,17,18,19,20,21,22]. Thermodynamic basis for generalized equations for heat transport [23,24,25,26,27,28,29,30,31,32,33,34,35] has been provided from non-equilibrium thermodynamics with internal variables and extended thermodynamics (see References [36,37,38,39,40,41,42,43,44,45,46,47,48,49,50,51,52,53,54,55,56,57,58,59,60,61,62,63,64,65,66,67,68]). Internal variables are a powerful tool for modeling complex media. Some remarks about the introduction of the internal variables and some versions of non-equilibrium thermodynamics will be made in Section 2.

In Reference [27], a “universal” approach was developed for heat rigid conductors, assuming that the entropy function depends on the internal energy and on a single vectorial internal variable quadratically. This internal variable may be a function of several microscopic variables of odd and even type with respect to the time reversibility. A generalized form for the entropy current is postulated and a generalized ballistic-conductive heat transport equation is obtained, which illustrates the universal character of the used approach.

In Reference [28], in the case of one-dimensional isotropic rigid heat conductors, assuming a quadratic dependence of the entropy density on two additional fields, the heat flux $J^{\left(q\right)}$ and a second-order tensor Q as internal variable, and a generalized form for the entropy flux, a generalized ballistic-conductive heat transport law is obtained, from which the special cases of Guyer–Krumhansl, Cahn–Hilliard-type, Jeffreys-type, Maxwell–Cattaneo–Vernotte and Fourier heat equations were derived. In Reference [33], the three-dimensional case was treated and both cases when the tensorial internal variable has odd parity or even parity were studied.

In Reference [9], an extended thermodynamic model for heat transfer at continuum scale and sub-continuum scale is given. At nanoscale, a two-fluid ballistic-diffusive hydrodynamic model is formulated, assuming that two types of heat carriers (phonons) coexist: the diffusive phonons, colliding with the core of the medium, according to the Maxwell–Vernotte–Cattaneo equation, and the ballistic phonons, colliding with the boundaries of the medium, whose motion is governed by the Guyer–Krumhansl equation. This ballistic-diffusion model was initially introduced by Chen in Reference [7], within the framework of the kinetic theory, wherein the distribution function is split into two contributions for diffusive phonons and for ballistic phonons. In this regard, see also Reference [53].

In Reference [34], assuming that the entropy function depends on the internal energy, and two internal variables, a vector and a second rank tensor, the total heat flux was split in two parts: a contribution obeying Fourier law and another one governed by the Maxwell–Vernotte–Cattaneo equation.

Several different thermodynamic formalisms may lead to slightly different versions of the Guyer–Krumhansl equation. This diversity may be useful when comparing different solutions, or may suggest new terms which could be relevant in particular problems.

As the motivation of this paper, we want to include the thermal vortices of phonons [69,70], arising in nonlinear phonon hydrodynamics and in GENERIC (general equation for non-equilibrium reversible-irreversible coupling) [71,72]. However, we also want to generalize the reflection on the local use variables (either the heat flux and its respective flux or the vectorial and tensorial variables) in non-local situations a little bit. Indeed, in the mentioned references, the non-locality is reflected in the evolution equations, but not in the definition of the basic variables themselves.

The paper is organized as follows. In Section 2, we recall some concepts regarding the internal variables and some different types of non-equilibrium thermodynamics, among them the Gyarmati wave approach [41], in whose frame we work in this paper. In Section 3, we introduce some fundamentals about the Gyarmati wave theory (see also [50]). In Section 4, Section 5 and Section 6, we review the thermodynamic formalism of the heat transport in rigid solids, with a polar vectorial internal variable, having odd parity. The phenomenological equations in the anisotropic and isotropic cases with the Onsager–Casimir relations and the entropy production are derived. In Section 7, we work out a generalized Guyer–Krumhansl heat transfer equation for isotropic media and in Section 8 and Section 9 we identify the internal variable with particular vector fields, deriving the corresponding heat transport laws and special cases of some of them. In Section 10, we discuss the relevant role of the entropy flux in non-local formulations, assuming that the entropy depends on the internal energy density, the heat flux and a tensorial internal variable. We also consider the case where the tensorial variable may have a symmetric part and an antisymmetric part, and explore how these parts, in the steady state, may be, respectively, related to the symmetric part and the antisymmetric part of the gradient of the heat flux.

We have also provided a wide bibliography on internal variables and some historical views of their use in non-equilibrium thermodynamics. We have thought that this bibliographic exercise, which we have carried out for our particular reflection, could also be of interest for young researchers interested in internal variables but not aware of their multiple aspects nor of the historical evolution of their use.

## 2. Some Remarks about the Internal Variables and Some Versions of Non-Equilibrium Thermodynamics

A treatment concerning the history of the introduction of internal variables in non-equilibrium thermodynamics, their role and their definition can be found in References [43,50,57,60,61,63,64,65,67]. Internal variables are a powerful tool to model complex media. Internal variables are different from dynamic degrees of freedom. The internal degrees of freedom obey their own balance equations, the internal variables do not [65,67]. In some papers, no distinction is made between the internal variable and the internal degree of freedom. In Reference [57], the distinction is made between an internal variable, which is measurable, and a hidden degree of freedom (a variable whose physical nature is unknown) which is not measurable.

Some local macroscopic internal variables describe the internal structure of a medium, which can present point defects inside the crystalline lattice, due to the lack of atoms, dislocation lines that disturb the periodicity of the lattice, porous channels, inhomogeneity and heterogeneity. They can also describe partial specific polarizations or partial specific magnetizations in a electromagnetic medium, which are parts of the total specific polarization or of the total specific magnetization of the medium, due to different types of irreversible microscopic phenomena, that give rise to the dielectric relaxation or to the magnetic relaxation. Furthermore, assuming that irreversible microscopic phenomena give rise to inelastic strains (such as slips or dislocations) it is possible to describe these phenomena splitting the total strain in partial strain tensors and to formulate a theory for mechanical relaxation phenomena.

Some of these variables are polar vectors, such as the specific polarizations $p^{\left(i\right)}$, or axial vectors such as the specific magnetizations $m^{\left(i\right)}$ (*i* = 1, 2, …, *n*) (see [73,74] and also [75,76]), partial heat fluxes $J^{\left(i\right)}$ (*i* = 0, 1, 2, …, *n*), contributions of the total heat flux $J^{\left(q\right)}$ [42], others are tensorial variables such as the partial inelastic strain tensors $\varepsilon_{\alpha \beta }^{\left(i\right)}\;\;$ (*i* = 1, 2, …, *n*; α,β=1,2,3), (with α and β spatial coordinates) contributions of the total strain tensor in a medium [77,78,79,80], the dislocation core tensor $a_{ij}$, with *i*, *j* = 1, 2, 3 spatial coordinates, á la Maruszewski [81] or the dislocation tensor introduced by the authors [82] (see also [83,84]), analogous to the tensor defined to describe the quantized vortices in turbulent superfluid Helium II [85], the porosity tensor $r_{ij}$ á la Kubik [86] (see also [87,88,89]), the inhomogeneity-grain density and the anisotropy-grain tensor both defined by Maruszewski [90] (see also [91]). Furthermore, the trace of the dislocation tensor is an internal scalar variable and describes the density of local dislocation lines at a point of the medium. There exist several other internal variables describing the internal structure of the medium.

In some formalisms, the internal variable is eliminated and does not appear in the final results, while in others there is a relaxation equation for this variable and it appears in the final results, as for the dislocation tensor or the porosity tensor and others.

In Reference [63], the internal variables are introduced axiomatically in non-equilibrium thermodynamics by seven fundamental concepts, among them the concepts establishing that “the internal variables may be included in the state space or may be not”, “in equilibrium the internal variables become dependent on the variables of the equilibrium subspace, but they are not determined by them, i.e., the equilibrium subspace is many-valued”, “additional rate equations for internal variables join the balance equations and constitutive equations”, and “internal variables need a model or a (microscopic or molecular) interpretation”.

In this paper, within the framework of the Gyarmati wave approach [41], we introduce a non-local internal variable to model special thermal motions of phonons. It is called in a general way ζ, to develop a general model, but after obtaining the results, its identification takes place, as in the paper [42], where the internal variable is not eliminated from the formalism but an interpretation of it is given. There are many schools of non-equilibrium thermodynamics [45,47,48,49,56,62,92].

Limiting ourselves to the theories in continuum field formulation, two classes of theories can be distinguished with respect to the general relation between the heat flux $J^{\left(q\right)}$ and the entropy flux density $J^{\left(s\right)}=\frac{J^{\left(q\right)}}{T}+k$, where k is an additional term called extra entropy flux density [44,49,93]. The theories where k is null, k=0, are called *Clausius–Duhem theories*. The choice of the state space of the “basic” fields together with different basic assumptions classify special versions of non-equilibrium thermodynamic theories.

In classical irreversible thermodynamics (CIT), the state space is the equilibrium subspace. This hypothesis is called the local equilibrium hypothesis, thus there are no gradients and time derivatives, nor fluxes in the state space. The entropy flux density is given by $J^{\left(s\right)}=\frac{J^{\left(q\right)}}{T}$ in the case of one component material or $J^{\left(s\right)}=\frac{1}{T}\left(J^{\left(q\right)}-\sum_{l=1}^{n}\mu^{\left(l\right)}J_{diff}^{\left(l\right)}\right)$ in the case where a medium consist of *n* components, with $J_{diff}^{\left(l\right)}$ the *l*-th diffusive flux and $\mu^{\left(l\right)}$ the thermodynamic or chemical potential of the component *l*-th. Furthermore, in non-equilibrium, the time rate of the entropy satisfies the Gibbs equation. The entropy production $\sigma^{\left(s\right)}$ is given by a sum of products of fluxes $J_{i}$ and forces $X_{i}$, $\sigma^{\left(s\right)}=J_{i}X_{i}$. Fluxes and forces are connected by linear constitutive equations, $J_{i}=L_{ik}X_{k}$, so that the entropy production is a bilinear form, $\sigma =L_{ik}X_{i}X_{k}$, where the Einstein sum convention regarding the repeated indices is used. The Onsager reciprocity relations state that the phenomenological coefficients relating thermodynamic fluxes to thermodynamic forces are symmetrical, i.e., $L_{ij}=L_{ji}$ [36,37,56].

In rational thermodynamics (RT) in the state space non-equilibrium variables are included, they can be gradients, time derivatives. It is assumed that the entropy and the temperature are primitive concepts, i.e., nothing is said about how to define (or to measure) these quantities in non-equilibrium. The fields of specific entropy *s*, the entropy flux density $J^{\left(s\right)}$ and entropy production density $\sigma^{\left(s\right)}$ are assumed as constitutive quantities (i.e., functions of the state space fields), which have to satisfy the dissipation inequality (local in time)

(1) $$\sigma^{\left(s\right)}=\rho \;\frac{ds}{dt}\;+\;\nabla \cdot J^{\left(s\right)}⩾0,$$

where ρ is the mass density field of the body, d/dt is material time derivative, defined by $\;\frac{d}{dt}\;=\;\frac{\partial }{\partial t}\;+\;v_{i}\;\frac{\partial }{\partial x^{i}}\;$, with $\frac{\partial }{\partial t}$ the partial time derivative, $v_{i}$ is the velocity field, and the symbol “∇·” denotes the divergence operator.

Indeed, the law (1) is the second law of thermodynamics that, with its amendment: “Except in equilibrium, there are no reversible process directions in the state space”, states that all local solutions of the balance equations have to satisfy the dissipation inequality (1), [45,47,48,49,56,94,95,96]. The inequality (1) is exploited by Coleman/Noll [97,98] or Liu procedure [99]. The principle of equipresence states that all constitutive quantities depend on the same set of variables of the state space. There are other material axioms restricting the constitutive mappings: material frame indifference, objectivity, and standard frame dependence [45,62,100].

In extended thermodynamics (ET) [55,56,58,62], in the state space there are present dissipative fluxes but no gradients or time derivatives. We distinguish two types of extended thermodynamics, which differ mainly in exploiting the dissipation inequality: the rational extended thermodynamics (RET), that is a further development of the rational thermodynamics, where the dissipation inequality is exploited by using the Liu or Coleman and Noll technique; the extended irreversible thermodynamics (EIT), that, starting from CIT, exploits the dissipation inequality as in CIT. Additional balance equations for dissipative fluxes are needed.

The Gyarmati wave approach, considers dissipative fluxes in the state space and a quadratic dependence of the entropy density on the added fields in the state space and uses the CIT procedures, see Section 3.

Another approach of the non-equilibrium thermodynamics is GENERIC [72,101,102,103], where the evolution equation of a state variable consists of a reversible part and an irreversible part. The reversible evolution is Hamiltonian, i.e., it is generated by a Poisson bracket and by the energy, the irreversible part is generated by a dissipation potential and by the entropy.

There exist other important different types of thermodynamics such as the endoreversible thermodynamics, the mesoscopic theory, the kinetic theory, the variational formulations and others [48,56,62].

## 3. Gyarmati Wave Approach: Fundamentals

In this paper, we treat the heat transfer phenomena in solid media within the framework of Gyarmati wave theory [41], where, as Verhás commented, “the current densities regarding transport processes have inertia and hence the part of the internal energy of the medium is a kinetic energy attributable to them” [50].

This section deals with the fundamental concepts and assumptions of this approach, giving some examples. The standard Cartesian tensor notation in a rectangular coordinate system is used and the equations governing the behavior of a heat-conducting rigid medium are considered in a current configuration $K_{t}$.

We consider the following balance equations:

the *mass conservation law* given by

(2) $$\frac{d\rho }{dt}\;+\rho \;\nabla \cdot v\;=\;0.$$

the *internal energy balance equation* in the form:

(3) $$\rho \;\frac{du}{dt}\;+\;\nabla \cdot J^{\left(q\right)}\;=\;0\;,$$

where *u* is the internal energy density (the internal energy per unit mass), $J^{\left(q\right)}$ is the heat flux vector, the current density of the internal energy, the heat source is disregarded and $\nabla \cdot J^{\left(q\right)}=tr\left(\nabla J^{\left(q\right)}\right)$, with “∇” as the symbol indicating the gradient operator; and the *entropy inequality* given by (1).

In this thermodynamic approach, to model phenomena beyond the local equilibrium, the specific entropy *s* of the medium is not function only on the local equilibrium state variables, but also comprehends additive contributions depending, in an homogeneous quadratic way, on vectorial, tensorial variables, and current densities, that describe the rate of the processes. In particular, in Reference [41] (see also Reference [50]), the specific entropy has the form

(4) $$s=s^{eq}\left(u\right)-\frac{1}{2}m{\left(J^{\left(q\right)}\right)}^{2},$$

where $s^{eq}\left(u\right)$ is the equilibrium entropy function and *m* is a material coefficient characterizing the inertia of the heat current density. Furthermore, m>0 by virtue of the concavity of the entropy function. The following quantities are defined:

(5) $$\frac{1}{T}=\;\frac{\partial s}{\partial u}=\frac{ds^{\left(eq\right)}}{du},\;\frac{\partial s}{\partial J_{i}^{\left(q\right)}}=\;-\;mJ_{i}^{\left(q\right)}\;\left(i=1,2,3\right),$$

where *T* is the equilibrium temperature.

By virtue of Equation (3) and the entropy balance Equation (1), with the entropy flux $J^{\left(s\right)},$ defined as in CIT, $J^{\left(s\right)}=\frac{1}{T}J^{\left(q\right)}$, using the procedures of the classical irreversible thermodynamics, the Maxwell–Cattaneo equation, describing high frequency processes, is obtained in the following form:

(6) $$\tau \frac{dJ^{\left(q\right)}}{dt}+J^{\left(q\right)}=-\lambda \nabla T,$$

with $\tau =\lambda mT^{2}$ the relaxation time of the heat flux and λ the heat conductivity coefficient.

The Gyarmati theory is a generalization of the classical irreversible thermodynamics and it was formulated on the basis of Onsager and Machlup approach for adiabatically closed systems, having a “kinetic energy” [104].

The same procedure is used in Reference [42], where a specific entropy is considered in the form

(7) $$s=s^{eq}\left(u\right)-\frac{1}{2}\sum_{k=1}^{n}{\left(\beta^{\left(k\right)}\right)}^{2},$$

where $\beta^{\left(k\right)}$ (*k* = 1, 2, …, *n*) are vectorial variables, called dynamic degrees of freedom, introduced in the state space $C=C(u,\beta^{\left(1\right)},\beta^{\left(2\right)},\ldots ,\beta^{\left(n\right)})$.

Defining $\frac{1}{T}=\;\frac{\partial s}{\partial u},\;$ $\frac{\partial s}{\partial \beta_{i}^{\left(k\right)}}=-\beta_{i}^{\left(k\right)}\;\left(k=1,2,\ldots ,n;\;i=1,2,3\right)$, using the entropy balance (1), with the entropy flux $J^{\left(s\right)}$ given by $J^{\left(s\right)}=\frac{1}{T}J^{\left(q\right)}$, and the internal energy balance Equation (3), by virtue of the CIT procedures, the following phenomenological equations are obtained in the isotropic case:

(8) $$J^{\left(q\right)}=-\lambda \nabla \;T-\rho \sum_{k=1}^{n}\mu^{\left(q)(k\right)}\frac{d\beta^{\left(k\right)}}{dt},\;\beta^{\left(k\right)}=-\mu^{\left(k)(q\right)}\nabla \;T-\rho \alpha^{\left(k\right)}\frac{d\beta^{\left(k\right)}}{dt}\;\left(k=1,2,\ldots ,n\right).$$

By the identifications: $\;J^{\left(0\right)}=-\lambda^{\left(0\right)}\nabla \;T,\;J^{\left(k\right)}=\frac{\mu^{\left(q)(k\right)}}{\alpha^{\left(k\right)}}\beta^{\left(k\right)},$ the following results are derived [42]:

(9) $$J^{\left(q\right)}=J^{\left(0\right)}+\sum_{k=1}^{n}J^{\left(k\right)},\;\;\tau^{\left(k\right)}\frac{dJ^{\left(k\right)}}{dt}+J^{\left(k\right)}=-\lambda^{\left(k\right)}\nabla \;T\;\left(k=1,2,\ldots ,n\right),$$

where $\lambda -\sum_{k=1}^{n}\frac{\mu^{\left(k)(q\right)}\mu^{\left(q)(k\right)}}{\alpha^{\left(k\right)}}=\lambda^{\left(0\right)}≧0,\;\mu^{\left(i)(q\right)}=T^{2}\mu^{\left(q)(i\right)},\;\alpha^{\left(k\right)}>0,\;\tau^{\left(k\right)}=\rho \alpha^{\left(k\right)},\;\lambda^{\left(k\right)}=T^{2}\frac{{\left(\mu^{\left(k)(q\right)}\right)}^{2}}{\alpha^{\left(k\right)}}\;\left(k=1,2,\ldots ,n\right).$

Equation (9) establishes that the heat flux $J^{\left(q\right)}$ is given by n+1 contributions: $J^{\left(0\right)}$ governed by Fourier constitutive equation, valid at low frequencies and large space scales, and the *n* contributions $J^{\left(k\right)}$ governed by Maxwell–Cattaneo equations, describing high frequency processes. $\tau^{k}$ is the relaxation time of the partial heat flux $J^{\left(k\right)}.$

In Reference [26], supposing that the entropy function depends besides the internal energy *u* also on the heat current $J^{\left(q\right)},$ in a way as suggested by the Gyarmati approach, a theory for non-local phenomena is formulated assuming a generalized entropy flux. The Guyer–Krumhansl heat transfer equation, with Fourier, Maxwell–Cattaneo–Vernotte as special cases, and the Cahn–Hilliard heat transport equation are derived. In Reference [29], the second sound, the wave-like propagation of heat is investigated at low temperatures, and assuming a quadratic dependence of the entropy density on the heat flux $J^{\left(q\right)}$ and a second order tensorial internal variable Q, postulating a generalized form for the entropy flux, using the Nyíri multipliers [105], a generalized ballistic-conductive heat transport model is obtained.

## 4. A Model Describing Special Thermal Motions of Phonons

The phonon hydrodynamics describes the heat transfer at sub-continuum scale, when the motion of the phonons (heat carriers) is taken into consideration. At nanoscale, for instance, we can consider the diffusive phonons, that undergo multiple collisions with the core of the medium, whose motion is governed by the Maxwell–Vernotte–Cattaneo equation, and the ballistic phonons, colliding with the walls of the medium, whose motion is described by the Guyer–Krumhansl equation [2,7,9,53]. In this paper, we suppose that particular thermal motions of the phonons, such as the thermal vortices [69,70], can be described by a non-local macroscopic vectorial internal variable ζ, of which we give some interpretations in Section 8 and Section 9. We work within the framework of the Gyarmati approach. Our objective is to derive generalized Guyer–Krumhansl evolution equations for the heat flux. Thus, if *u* is the specific internal energy, the thermodynamic state space is chosen as follows:

(10) C=C(u,ζ),

and the specific entropy *s* depends on the specific internal energy *u* and on the internal variable ζ, i.e.,

(11) s=s(u,ζ).

We define

(12) $$\left\{\begin{array}{cc} \frac{1}{T}\; & =\;\frac{\partial s}{\partial u}\\ \frac{\partial s}{\partial \zeta_{i}}\; & =\;-\;\eta_{ij}\zeta_{j}, \end{array}\right.$$

where *T* is the equilibrium temperature (the absolute temperature) and $\eta_{ij}$ are constitutive coefficients characterizing the material under consideration (they are called inductivities).

The mass conservation law and the internal energy balance equation are given by (2) and (3). We assume an entropy function having the form:

(13) $$s\left(u,\zeta \right)\;=\;s^{\left(eq\right)}\left(u\right)\;-\frac{1}{2}\eta_{ij}\zeta_{i}\zeta_{j}\;\;\left(i,j=1,2,3\right),$$

where $s^{eq}\left(u\right)$ is the equilibrium entropy function depending only on the internal energy. Furthermore, from (13) the coefficients $\eta_{ij}$ satisfy the following symmetry relations:

(14) $$\eta_{ij}=\eta_{ji}\;\;\left(i,j=1,2,3\right).$$

Thermodynamic stability requires that the inductivity tensors $\eta_{ji}$ are positive definite (see Appendix A), and we assume that they are constant. Using Equations (12) and (13), we obtain the following Gibbs relation:

(15) $$T\;ds\;=\;du\;-T\;\eta_{ij}\zeta_{i}\;d\zeta_{j}.$$

From (15), it follows that the time derivative of the entropy *s* has the form

(16) $$\varrho T\;\frac{ds}{dt}\;=\;\varrho \;\frac{du}{dt}\;-\varrho T\;\eta_{ij}\zeta_{i}\;\frac{d\zeta_{j}}{dt}.\;$$

Using Equation (3), from (16) the entropy balance is obtained in the form (1), where the entropy flux $J^{\left(s\right)}$ is given by $J^{\left(s\right)}=\frac{1}{T}J^{\left(q\right)}$, and the entropy production has the following form:

(17) $$\sigma^{\left(s\right)}\;=\;T^{-1}\;\left[-T^{-1}\;J_{i}^{\left(q\right)}\frac{\partial T}{\partial x^{i}}\;-\;\varrho \;T\;\eta_{ij}\;\zeta_{i}\;\frac{d\zeta_{j}}{dt}\;\right]\geq 0.$$

From (17), it is seen that the entropy production is composed additively of two contributions, the first term is due to the heat conduction and the second term is connected with the entropy production due to the internal variable field.

Here, we consider the case of rigid conductors at rest, so that the material time derivative $\frac{d}{dt}$ is equal to the partial time derivative $\frac{\partial }{\partial t}$.

## 5. Phenomenological Equations

The entropy production (17) is a bilinear form composed of a sum of terms, where each term is a product of a flux and an affinity (or thermodynamic force) conjugate to the flux [39,57]. We choose as fluxes $J_{i}^{\left(q\right)}$ and $\varrho T\eta_{ij}\frac{\partial \zeta_{j}}{\partial t}$ and as affinities $-T^{-1}\frac{\partial T}{\partial x^{i}}$ and $-\zeta_{i}$. According to the procedures of non-equilibrium thermodynamics, we derive the following phenomenological equations for anisotropic media, where the irreversible fluxes are linear functions of the affinities

(18) $$J_{i}^{\left(q\right)}=L_{ij}^{\left(q)(q\right)}\left(-T^{-1}\frac{\partial T}{\partial x^{j}}\right)+L_{ij}^{\left(q)(1\right)}(-\zeta_{j})\;,$$

(19) $$\varrho T\eta_{ij}\frac{\partial \zeta_{j}}{\partial t}=L_{ij}^{\left(1)(q\right)}\left(-T^{-1}\frac{\partial T}{\partial x^{j}}\right)+L_{ij}^{\left(1)(1\right)}(-\zeta_{j})\;.$$

Equation (18) is a generalization of Fourier law and Equation (19) is the phenomenological equation for the irreversible processes described by the internal variable ζ. The quantities $L_{ij}^{\left(q)(q\right)}$, $L_{ij}^{\left(q)(1\right)}$, $L_{ij}^{\left(1)(q\right)}$, $L_{ij}^{\left(1)(1\right)}$, that occur in (18) and (19) are phenomenological tensors and are supposed constant. $L_{ij}^{\left(q)(q\right)}$ is the second order tensor of the heat conductivity, $L_{ij}^{\left(q)(1\right)}$ and $L_{ij}^{\left(1)(q\right)}$ are second order tensors connected with the influence of the heat flux on the field of the internal variable and vice versa and $L_{ij}^{\left(1)(1\right)}$ is a second order tensor connected with the irreversible phenomena due to the field ζ. The macroscopic quantities, which occur in a phenomenological theory, from the microscopic point of view, can depend on the velocity of the microscopic particles, distinguishing them in even and odd functions if they are even or odd functions of the speed of these particles. From the macroscopic point of view, we distinguish the macroscopic quantities in even and odd functions, when they are even or odd under time reversal [39,56,57,72].

The heat flux $J^{\left(q\right)}$ is an odd function and we assume that the internal variable ζ is an *odd polar vector*; $T^{-1}grad\;T$ and the time derivative of ζ, $\frac{\partial \zeta }{\partial t}$ are even functions. Hence, we have the following Onsager–Casimir reciprocity relations for the phenomenological coefficients

(20) $$L_{ij}^{\left(q)(q\right)}=L_{ji}^{\left(q)(q\right)}\;,\;L_{ij}^{\left(q)(1\right)}=-L_{ji}^{\left(1)(q\right)}\;,\;L_{ij}^{\left(1)(1\right)}=L_{ji}^{\left(1)(1\right)}\;.$$

Relations (20) reduce the number of independent components of the phenomenological tensors, that are polar tensors. Substituting the laws (18) and (19) into (17), by virtue of relations (20), one has

(21) $$T\;\sigma^{\left(s\right)}\;=\;T^{-2}L_{ij}^{\left(q)(q\right)}\;\frac{\partial T}{\partial x^{i}}\;\frac{\partial T}{\partial x^{j}}\;+L_{ij}^{\left(1)(1\right)}\;\zeta_{j}\zeta_{i}.$$

From (21) it is seen that the entropy production in a non-negative bilinear quadratic form in the components of the gradient of the temperature and the components of the internal variable. In Appendix A, we give a matrix representation of $\sigma^{\left(s\right)}$ in the form: $\sigma^{\left(s\right)}=X_{\alpha }L_{\alpha \beta }X_{\beta }$, being $X_{\alpha }$, $X_{\beta }$ and $L_{\alpha \beta }$ suitable matrices see (A2)–(A4). Some inequalities are derived for the components of the phenomenological tensors, resulting from the fact that all the elements of the main diagonal of the symbolic matrix $L_{\alpha \beta }$ associated with the bilinear form (21) must be non-negative, representing a condition (only necessary) for the semi-definiteness of the matrix $L_{\alpha \beta }$. In particular, we have obtained

(22) $$L_{ii}^{\left(q)(q\right)}\geq 0,\;L_{ii}^{\left(1)(1\right)}\geq 0,\;\left(i,j=1,2,3\right).$$

Furthermore, other relations can be derived from the non-negativity of the major minors of $L_{\alpha \beta }$, coming from Sylvester’s criterion, that represents a necessary and sufficient condition for the semi-definiteness of the matrix $L_{\alpha \beta }$.

## 6. Isotropic Case

The existence of spatial symmetry properties in the media under consideration may simplify the form of the phenomenological equations in such way that the Cartesian components of the fluxes do not depend on all Cartesian components of the thermodynamic forces. This statement is called Curie symmetry principle.

In this Section, we consider perfect isotropic media for which the symmetry properties are invariant under orthogonal transformations with respect to all rotations and inversions of the axes frame.

In this case, polar tensors of order two keep the form $\;L_{ij}=L\delta_{ij},\;$ where L is a scalar.

Thus, in the isotropic case, $\eta_{ij}=\eta \delta_{ij}$ and the entropy function (13) has the following form:

(23) $$s\left(u,\zeta \right)=s^{\left(eq\right)}\left(u\right)-\frac{1}{2}\eta \zeta^{2}.$$

Defining

(24) $$\left\{\begin{array}{cc} \frac{1}{T}\; & =\;\frac{\partial s}{\partial u}\\ \frac{\partial s}{\partial \zeta_{i}}\; & =\;-\;\eta \zeta_{i}, \end{array}\right.$$

from (23) and (24), the following Gibbs relation is derived:

(25) $$T\;ds\;=\;du\;-T\;\eta \zeta_{i}\;d\zeta_{i}.$$

From (3) and (25), the entropy balance is obtained in the form (1), where $J^{\left(s\right)}=\frac{1}{T}J^{\left(q\right)}$, and the entropy production has the following form:

(26) $$\sigma^{\left(s\right)}\;=\;T^{-1}\;\left[-T^{-1}\;J_{i}^{\left(q\right)}\frac{\partial T}{\partial x^{i}}\;-\;\varrho \;T\;\eta \zeta_{i}\;\frac{d\zeta_{i}}{dt}\;\right]\geq 0.$$

From (26), following the procedures of non-equilibrium thermodynamics, the phenomenological equations in the isotropic case have the form (see also (18) and (19))

(27) $$J_{i}^{\left(q\right)}=L^{\left(q)(q\right)}\left(-T^{-1}\frac{\partial T}{\partial x^{i}}\right)+L^{\left(q)(1\right)}\left(-\zeta_{i}\right),$$

(28) $$\varrho \eta T\frac{\partial \zeta_{i}}{\partial t}=L^{\left(1)(q\right)}\left(-T^{-1}\frac{\partial T}{\partial x^{i}}\right)+L^{\left(1)(1\right)}\left(-\zeta_{i}\right).$$

The Onsager–Casimir reciprocity for the phenomenological coefficients are

(29) $$L^{\left(q)(q\right)}=L^{\left(q)(q\right)},\;L^{\left(q)(1\right)}=-L^{\left(1)(q\right)},\;L^{\left(1)(1\right)}=L^{\left(1)(1\right)},$$

see also (20), being in the isotropic case

$$L_{ij}^{\left(q)(q\right)}=L^{\left(q)(q\right)}\delta_{ij},\;L_{ij}^{\left(q)(1\right)}=L^{\left(q)(1\right)}\delta_{ij},\;L_{ij}^{\left(1)(q\right)}=L^{\left(1)(q\right)}\delta_{ij},\;L_{ij}^{\left(1)(1\right)}=L^{\left(1)(1\right)}\delta_{ij}.$$

Substituting the laws (27) and (28) into Equation (26), by virtue of the relations (29), one has

(30) $$T\;\sigma^{\left(s\right)}\;=\;T^{-2}L^{\left(q)(q\right)}{\left(\frac{\partial T}{\partial x^{i}}\right)}^{2}+L^{\left(1)(1\right)}{\left(\zeta_{i}\right)}^{2}.$$

The entropy production (30) is a positive definite quadratic form in the components of the gradient of the temperature and the components of the internal variable, thus we have (see Appendix A)

(31) $$\;\;L^{\left(q)(q\right)}\geq 0,\;L^{\left(1)(1\right)}\geq 0.$$

Furthermore, the thermodynamic stability requires that

(32) η>0

(see Appendix B). We assume that η is constant.

## 7. A Generalized Guyer-Krumhansl Heat Transfer Equation in Rigid Media

In this section, we derive a generalized Guyer–Krumhansl equation for the heat transport in rigid media at rest. First, we calculate the partial time derivative of Equation (27) multiplied by $\varrho T\eta L^{\left(q)(q\right)},$ obtaining

(33) $$\varrho T\eta L^{\left(q)(q\right)}{\dot{J}}_{i}^{\left(q\right)}=\varrho T\eta {\left(L^{\left(q)(q\right)}\right)}^{2}\left[-T^{-1}\frac{\partial \dot{T}}{\partial x^{i}}+T^{-2}\dot{T}\frac{\partial T}{\partial x^{i}}\right]+L^{\left(q)(q\right)}L^{\left(q)(1\right)}\left(-\varrho T\eta {\dot{\zeta }}_{i}\right),$$

where the upper dot denotes the partial time derivative.

Furthermore, Equation (27), multiplied by $L^{\left(1)(q\right)},$ and added to the Equation (28), multiplied by $L^{\left(q)(q\right)},$ gives

$$L^{\left(1)(q\right)}J_{i}^{\left(q\right)}+L^{\left(q)(q\right)}\varrho \eta T{\dot{\zeta }}_{i}=$$

(34) $$+2L^{\left(1)(q\right)}L^{\left(q)(q\right)}\left(-T^{-1}\frac{\partial T}{\partial x^{i}}\right)+\left(L^{\left(1)(q\right)}L^{\left(q)(1\right)}+L^{\left(q)(q\right)}L^{\left(1)(1\right)}\right)\left(-\zeta_{i}\right).$$

From Equation (34), multiplied by $L^{\left(1)(q\right)}$, and Equation (33), we obtain

$$\varrho T\eta L^{\left(q)(q\right)}{\dot{J}}_{i}^{\left(q\right)}+{\left(L^{\left(1)(q\right)}\right)}^{2}J_{i}^{\left(q\right)}=\varrho T\eta {\left(L^{\left(q)(q\right)}\right)}^{2}\left[-T^{-1}\frac{\partial \dot{T}}{\partial x^{i}}+T^{-2}\dot{T}\frac{\partial T}{\partial x^{i}}\right]+$$

(35) $$+2{\left(L^{\left(1)(q\right)}\right)}^{2}L^{\left(q)(q\right)}\left(-T^{-1}\frac{\partial T}{\partial x^{i}}\right)+L^{\left(1)(q\right)}\left(L^{\left(1)(q\right)}L^{\left(q)(1\right)}+L^{\left(q)(q\right)}L^{\left(1)(1\right)}\right)\left(-\zeta_{i}\right),$$

i.e.,

(36) $$\tau_{q}{\dot{J}}_{i}^{\left(q\right)}+J_{i}^{\left(q\right)}=-\lambda \frac{\partial T}{\partial x^{i}}-\nu \frac{\partial \dot{T}}{\partial x^{i}}+\mu \dot{T}\frac{\partial T}{\partial x^{i}}+\gamma \zeta_{i},$$

with

$$\tau_{q}=\frac{\varrho T\eta L^{\left(q)(q\right)}}{{\left(L^{\left(1)(q\right)}\right)}^{2}},\;\lambda =2T^{-1}L^{\left(q)(q\right)}\;\nu =\frac{\varrho \eta {\left(L^{\left(q)(q\right)}\right)}^{2}}{{\left(L^{\left(1)(q\right)}\right)}^{2}},\;\mu =T^{-1}\frac{\varrho \eta {\left(L^{\left(q)(q\right)}\right)}^{2}}{{\left(L^{\left(1)(q\right)}\right)}^{2}},$$

(37) $$\gamma =\left(-L^{\left(q)(1\right)}+\frac{L^{\left(q)(q\right)}L^{\left(1)(1\right)}}{L^{\left(q)(1\right)}}\right)=\left(-L^{\left(q)(1\right)}-\frac{L^{\left(q)(q\right)}L^{\left(1)(1\right)}}{L^{\left(1)(q\right)}}\right),$$

where $\tau_{q}$ is the relaxation time of the heat flux, λ is the heat conductivity, $L^{\left(q)(q\right)}$, $L^{\left(1)(1\right)}$ and η are positive (see (31) and (32)) and we have taken into account that $L^{\left(q)(1\right)}=-L^{\left(1)(q\right)}$ (see relation (29)).

Furthermore, we have to suppose that in Equation (36), we have

(38) $$L^{\left(q)(q\right)}\neq 0\;\mathrm{and}\;L^{\left(q)(1\right)}=-L^{\left(1)(q\right)}\neq 0,$$

(see expressions of $\tau_{q},\lambda ,\nu ,\mu$ and γ in (37)).

From the expressions (37) we have the following relations among the coefficients $\tau_{q},\lambda ,\nu ,\mu$:

(39) $$\tau_{q}=\frac{\rho T^{2}\eta \lambda }{2{\left(L^{\left(q)(1\right)}\right)}^{2}},\;\nu =\frac{\rho T^{2}\eta \lambda^{2}}{4{\left(L^{\left(q)(1\right)}\right)}^{2}},\;\mu =\frac{\rho T\eta \lambda^{2}}{4{\left(L^{\left(q)(1\right)}\right)}^{2}},\;\nu =T\mu ,\;\nu =\frac{\tau_{q}\lambda }{2},\;\mu =\frac{\tau_{q}\lambda }{2T}.$$

To obtain generalized Guyer–Krumhansl evolution equations for the heat flux, we have to impose that γ is positive. From the first expression of γ on the right hand side in (37)${}_{5}$, we derive the following constraints concerning the phenomenological coefficients:

(40) γ>0if

(41) $$\;L^{\left(q)(q\right)}L^{\left(1)(1\right)}>{\left(L^{\left(q)(1\right)}\right)}^{2},\;\mathrm{and}\;\;L^{\left(q)(1\right)}>0,$$

or

(42) $$\;\;L^{\left(q)(q\right)}L^{\left(1)(1\right)}<{\left(L^{\left(q)(1\right)}\right)}^{2}\;\mathrm{and}\;L^{\left(q)(1\right)}<0.$$

From the second expression of γ on the right hand side in (37)${}_{5}$, we have

(43) γ>0if

(44) $$\;\;L^{\left(q)(q\right)}L^{\left(1)(1\right)}<{\left(L^{\left(1)(q\right)}\right)}^{2},\;\mathrm{and}\;\;L^{\left(1)(q\right)}>0,$$

or

(45) $$\;L^{\left(q)(q\right)}L^{\left(1)(1\right)}>{\left(L^{\left(1)(q\right)}\right)}^{2}\;\mathrm{and}\;L^{\left(1)(q\right)}<0.$$

The constraints (40)–(42) are equivalent to the constraints (43)–(45), taking into consideration that ${\left(L^{\left(q)(1\right)}\right)}^{2}={\left(L^{\left(1)(q\right)}\right)}^{2}$, being $L^{\left(q)(1\right)}=-L^{\left(1)(q\right)}$.

In the Section 8, Section 9 and Section 10, we give some proposals for the internal variable.

## 8. Proposal I for the Internal Variable: ζ = $\alpha (J^{\left(q\right)}+R^{2}\Delta J^{\left(q\right)})$

Up to now, we have worked with ζ in general terms, for which we have not provided any particular interpretation, in such a way that the calculations are more manageable. Here, we are interested in the Guyer–Krumhansl equation, going beyond the Maxwell–Cattaneo one because of the presence of non-local terms having the form of a Laplacian of the heat flux times the square of the mean free path. Because of this, as an illustration of our proposal to use non-local variables we assume, for instance, that instead of taking the heat flux as in the paper [41], or a vector proportional to it, one can take the more general expression

(46) $$\zeta =\alpha (J^{\left(q\right)}+R^{2}\Delta J^{\left(q\right)}),$$

where the symbol “Δ” indicates the Laplacian operator. Note that the variable ζ, that we are proposing is not local, i.e., it contains Laplacian terms times the square of the observation length *R*. When such observation length is small enough, i.e., when the system is homogeneous at the scale of *R*, this non-local term in the variable may be neglected, and we obtain a theory with local variables following non-local evolution equations. However, if the system is inhomogeneous at the scale of *R*, this term could be used to reflect the degree of local inhomogeneity in the system, giving a correction to the average value of $J^{\left(q\right)}$. From a statistical point of view, one could say that the first term in ζ is the average of $J^{\left(q\right)}$ in the elementary region *R*, and that the second term is related to the local variance of $J^{\left(q\right)}$ in such region. We think that such considerations are worthy of further exploration.

From Equation (36), with the relations (37)–(45), we have

(47) $$\tau_{q}{\dot{J}}^{\left(q\right)}+\left(1-\gamma \alpha \right)J^{\left(q\right)}=-\lambda \nabla \;T-\nu \nabla \;\dot{T}+\mu \dot{T}\nabla \;T+\gamma \alpha R^{2}\Delta J^{\left(q\right)}.$$

So that Equation (47) represents a generalized Guyer–Krumhansl evolution equation for the heat flux, we impose:

(48) γα>0,and(1−γα)>0,fromwhichα>0andα<1/γ.

From (46)–(48), we derive that for the validity of (47) we have to take into consideration in the choice of ζ the following constraint for α

(49) $$\zeta =\alpha \left(J^{\left(q\right)}+R^{2}\Delta J^{\left(q\right)}\right),\;\;\mathrm{with}\;\;\;0<\alpha <1/\gamma ,\;\;i.e.,\;\;0<\alpha <\frac{L^{\left(q)(1\right)}}{L^{\left(q)(q\right)}L^{\left(1)(1\right)}-{\left(L^{\left(q)(1\right)}\right)}^{2}}.$$

In (47), we see that from the option to choose the internal variable ζ in the form (49) we have obtained the presence of non-local terms having the form of a Laplacian of the heat flux times the quantity $\gamma \alpha R^{2}$ and a total heat flux $\left(1-\gamma \alpha \right)J^{\left(q\right)}$, which is given by two contributions. This last situation is contemplated several times in thermodynamics with internal variables, in the sense that a macroscopic physical quantity describing particular phenomena in complex media, with internal structure, is split in more parts to describe new phenomena (see for instance the case of studies on special mechanical, dielectric, magnetic effects [75,76]). Here, we are treating thermal phenomena. Thus, we obtain

(50) $$\tau_{q}{\dot{J}}^{\left(q\right)}+J^{\left(q\right)}=-\lambda \nabla \;T-\nu \nabla \;\dot{T}+\mu \dot{T}\nabla \;T+\gamma \Delta J^{\left(q\right)},$$

where we have continued to call $\tau_{q},\lambda ,\nu ,\mu ,\left(\gamma \alpha R^{2}\right)$ the coefficients present in (46) divided by (1−γα). Equation (48) is a generalized Guyer–Krumhansl heat equation.

## 9. Proposal II for the Internal Variable: ζ $=\nabla \nabla \cdot J^{\left(q\right)}$

In this section, in order to model special thermal motions in a heat conducting solid, we propose interpreting ζ as the gradient of the divergence of the heat flux $\nabla \nabla \cdot J^{\left(q\right)}$, which is a vector proportional to $\nabla \dot{u}.$ From the balance energy Equation (3), we have

(51) $$\nabla \nabla \cdot J^{\left(q\right)}\;=-\varrho \nabla \;\dot{u},$$

ϱ being supposed constant. Thus, the chosen internal variable describes a negative non-local variation of an instantaneous variation of *u*, i.e., $-\nabla (\frac{\partial u}{\partial t})$, or a negative instantaneous variation of a non-local variation of *u*, i.e., $-\frac{\partial (\nabla u)}{\partial t}$. From this definition of ζ, one obtains

(52) $$\zeta_{j}\;\overset{\mathrm{def}}{=}{\left(\nabla \;\nabla \cdot J^{\left(q\right)}\right)}_{j}\overset{\mathrm{def}}{=}{\left(\frac{\partial J_{i}^{\left(q\right)}}{\partial x_{i}}\right)}_{,j}=\frac{\partial^{2}J_{i}^{\left(q\right)}}{\partial x_{j}\partial x_{i}}=J_{i,ij}^{\left(q\right)}=J_{i,ji}^{\left(q\right)},$$

where a comma in lower indices indicates the spatial derivation and the indices assume values 1, 2, 3. Expression (52) can be reformulated as

(53) $$\zeta =\left(\nabla \nabla \cdot J^{\left(q\right)}\right)=\nabla \times \left(\nabla \times J^{\left(q\right)}\right)+\nabla \cdot \nabla J^{\left(q\right)}=\nabla \times \left(\nabla \times J^{\left(q\right)}\right)+\Delta J^{\left(q\right)},$$

where the symbol “×” indicates the vectorial product. By virtue of (53), Equation (36), with the relations (37)–(45), takes the following form

(54) $$\tau_{q}{\dot{J}}^{\left(q\right)}+J^{\left(q\right)}=-\lambda \nabla T-\nu \nabla \dot{T}+\mu \dot{T}\nabla T+\gamma \nabla \times \left(\nabla \times J^{\left(q\right)}\right)+\gamma \Delta J^{\left(q\right)}.$$

Equation (54) is a generalized heat equation for rigid heat conducting media, more general than the well-known Guyer–Krumhansl, when γ is positive, because in (54) there are the terms in $\nabla \dot{T}$ and $\dot{T}\nabla T$ and the term $\nabla \times (\nabla \times J^{\left(q\right)})$, describing effects of vortices of heat flux. In Fourier theory, the term $\nabla \times (\nabla \times J^{\left(q\right)})$ is zero because $J^{\left(q\right)}$ comes from a gradient, but in more general theories, and especially in phonon hydrodynamics, that term is in principle admissible, as it is in the usual hydrodynamics.

Note that the coefficient γ is proportional, in general, to some relaxation time times the square of a mean-free path of the heat carriers (phonons). Such a relaxation time is not necessarily the same as the relaxation time of the heat flux, but it is characteristic of the decay of $\nabla \nabla \cdot J^{\left(q\right)}$. This is logical because we have assumed that this quantity to be an internal variable, with a dynamics slightly different from that of $J^{\left(q\right)}$ itself.

From (54), it is possible to derive as particular cases some well-known equations, analyzed in the literature of heat transport. When the coefficients ν and μ can be disregarded, $\gamma =l^{2}$ (with *l* the mean free path of phonons) and the term in $\nabla \times (\nabla \times J^{\left(q\right)})$ is ignored, Equation (54) coincides with the *Guyer–Krumhansl* equation, i.e.,

(55) $$\tau_{q}{\dot{J}}^{\left(q\right)}+J^{\left(q\right)}=-\lambda \nabla T+l^{2}\Delta J^{\left(q\right)}.$$

If one ignores in (54) the terms in $\;\dot{T}\nabla T$, $\;\nabla \times (\nabla \times J^{\left(q\right)})$ and $\Delta J^{\left(q\right)}$, but not the other terms, it reduces to the analogous of the double lag equation proposed by Tzou [106], having the form

(56) $$\tau_{q}{\dot{J}}^{\left(q\right)}+J^{\left(q\right)}=-\lambda \nabla T-\tau \nabla \dot{T}.$$

In such an equation, one has two different relaxation times, $\tau_{q}$ and

(57) $$\tau =\frac{1}{2}\tau_{q}\lambda$$

(see expressions (39)) related to the lag in $J^{\left(q\right)}$ and to the lag in ∇T, respectively. From (57), we see that only both relaxation times $\tau_{q}$ and τ can be zero, not separately.

When $l^{2}$ is negligible, Equation (55) reduces to the following *Maxwell–Vernotte–Cattaneo* equation, describing second-sound phenomena (heat waves), and governing, at sub-continuum scale, the diffusive motion of the phonons, which are subjected to collisions with the core of the medium.

(58) $$\tau_{q}{\dot{J}}^{\left(q\right)}+J^{\left(q\right)}=-\lambda \nabla T.$$

It describes thermal signals having relaxation time $\tau_{q}$ and finite propagation velocity. In the case where in Equation (58) the relaxation time $\tau_{q}$ is negligible, it reduces to the *Fourier* equation $J^{\left(q\right)}=-\lambda \nabla T$, valid at low frequencies and large space scales.

## 10. The Role of the Entropy Flux in Non-Local Formulations

A usual way of dealing with non-local effects is related to the entropy flux, which has not appeared in our proposal in the former sections, and which we consider here for the sake of a more complete analysis. For instance, one may assume an entropy function $s(u,J^{\left(q\right)},Q)$, with Q a tensorial internal variable, having the following form (see [13,23,24,55,56,58,59]):

$$s\left(u,\zeta \right)=s^{\left(eq\right)}\left(u\right)-\frac{1}{2}\alpha_{1}{\left(J^{\left(q\right)}\right)}^{2}-\frac{1}{2}\alpha_{2}Q:Q,$$

where $\alpha_{1}$ and $\alpha_{1}$ are the inductivity constants, that the thermodynamic stability requires positive and the symbol “:” indicates double contraction, i.e., $Q:Q=Q_{ij}Q_{ij}.$ We define $\;\frac{1}{T}=\frac{\partial s}{\partial u},\;\frac{\partial s}{\partial J_{i}^{\left(q\right)}}=-\alpha_{1}J_{i}^{\left(q\right)},\;\frac{\partial s}{\partial Q_{ij}}=-\alpha_{2}Q_{ij}.\;$ We postulate the entropy flux in the form

(59) $$J^{\left(s\right)}=T^{-1}J^{\left(q\right)}-\beta_{1}Q\cdot J^{\left(q\right)},$$

where $\beta_{1}$ is a material coefficient, that conveniently represents the deviation of $J^{\left(s\right)}$ from the local equilibrium, and $Q\cdot J^{\left(q\right)}$ in components has the form $Q_{ij}J_{j}^{\left(q\right)}.$ Thus, one has

(60) $$ds=T^{-1}du-\alpha_{1}J^{\left(q\right)}\cdot dJ^{\left(q\right)}-\alpha_{2}Q:dQ,$$

from which, by virtue of (1), (3) and (59), we obtain the following entropy production:

$$\sigma^{s}=-\alpha_{1}J^{\left(q\right)}\cdot {\dot{J}}^{\left(q\right)}-\alpha_{2}Q:\dot{Q}+J^{\left(q\right)}\cdot \nabla T^{\left(-1\right)}-\beta_{1}\left[J^{\left(q\right)}\cdot \left(\nabla \cdot Q^{T}\right)+Q:{\left(\nabla J^{\left(q\right)}\right)}^{T}\right]=$$

(61) $$J^{\left(q\right)}\cdot \left[\nabla T^{\left(-1\right)}-\alpha_{1}{\dot{J}}^{\left(q\right)}-\beta_{1}\nabla \cdot Q^{T}\right]+Q:\left[-\alpha_{2}\dot{Q}-\beta_{1}{\left(\nabla J^{\left(q\right)}\right)}^{T}\right],$$

where we have denoted by the upper dot the partial time derivative $\frac{\partial }{\partial t}$, supposing the rigid conductor we are considering at rest, and we have taken into account that $\nabla \cdot \left(Q\cdot J^{\left(q\right)}\right)=J^{\left(q\right)}\cdot \left(\nabla \cdot Q^{T}\right)+Q:{\left(\nabla J^{\left(q\right)}\right)}^{T}),$ with $Q^{T}$ the transposed tensor of Q, ${\left(Q_{ji}\right)}^{T}=Q_{ij}.$

We assume the following linear relations between the thermodynamic fluxes $J^{\left(q\right)}$, Q and the forces $[\nabla T^{\left(-1\right)}-\alpha_{1}{\dot{J}}^{\left(q\right)}-\beta_{1}\nabla \cdot Q],$ $\left[-\alpha_{2}\dot{Q}-\beta_{1}J^{\left(q\right)}\right]$, as constitutive equations

(62) $$J^{\left(q\right)}=\lambda_{1}T^{2}\left[\nabla T^{\left(-1\right)}-\alpha_{1}{\dot{J}}^{\left(q\right)}-\beta_{1}\nabla \cdot Q\right],$$

(63) $$Q=\lambda_{2}\;\left[-\alpha_{2}\dot{Q}-\beta_{1}\nabla J^{\left(q\right)}\right],$$

with $\alpha_{1}$ and $\alpha_{2}$ proportional to the relaxation times of $J^{\left(q\right)}$ and Q, respectively. If $\alpha_{2}$ is very small with respect to $\alpha_{1}$ and it may be neglected, Q is proportional to $-\lambda_{2}\beta_{1}\nabla J^{\left(q\right)}$ and, introduced in (62), leads to

(64) $$J^{\left(q\right)}=-\lambda_{1}\nabla T-\tau {\dot{J}}^{\left(q\right)}+l^{2}\Delta J^{\left(q\right)},$$

with $\tau \equiv \lambda_{1}T^{2}\alpha_{1},$ $l^{2}\equiv \lambda_{1}\lambda_{2}T^{2}\beta_{1}^{2}$ and $\lambda_{1}$ and $\lambda_{2}$ positive. Since the fluxes are related to transfer from one point to another point, it is logical that they play an important role in non-local theories. Note that instead of $J^{\left(q\right)}$ in (60), one could have a vectorial internal variable J, which is usually interpreted a posteriori as proportional to $J^{\left(q\right)}$.

One could also generalize (59). Usually, it is considered that the internal variable Q is a symmetric tensor, but one may also consider, in more general terms, that Q has a symmetric part $Q^{s}$ plus an antisymmetric part $Q^{a}$, which may be related to an axial vector $Q^{\left(a\right)},$ which are independent variables in the formalism. Thus, we assume an entropy function having the following form: $s\left(u,J^{\left(q\right)},Q^{s},Q^{a}\right)=s^{\left(eq\right)}\left(u\right)-\frac{1}{2}\alpha_{1}{\left(J^{\left(q\right)}\right)}^{2}-\frac{1}{2}\alpha_{2}^{s}Q^{s}:Q^{s}-\frac{1}{2}\alpha_{2}^{a}Q^{a}:Q^{a},$ and an entropy flux $J^{\left(s\right)}$ having the form:

(65) $$J^{\left(s\right)}=T^{-1}J^{\left(q\right)}-\beta_{1}^{s}Q^{s}\cdot J^{\left(q\right)}-\beta_{1}^{a}Q^{a}\cdot J^{\left(q\right)}=T^{-1}J^{\left(q\right)}-\beta_{1}^{s}Q^{s}\cdot J^{\left(q\right)}-\beta_{1}^{a}Q^{a}\times J^{\left(q\right)},$$

being $Q^{a}\cdot J^{\left(q\right)}=Q^{\left(a\right)}\times J^{\left(q\right)}.$

The entropy differential may be written as

(66) $$ds=T^{-1}du-\alpha_{1}J^{\left(q\right)}\cdot dJ^{\left(q\right)}-\alpha_{2}^{s}Q^{s}:dQ^{s}-\alpha_{2}^{a}Q^{a}:dQ^{a},$$

having defined $\frac{1}{T}=\frac{\partial s}{\partial u},\;\frac{\partial s}{\partial J_{i}^{\left(q\right)}}=-\alpha_{1}J_{i}^{\left(q\right)},\;\frac{\partial s}{\partial Q_{ij}^{s}}=-\alpha_{2}^{s}Q_{ij}^{s},\;\frac{\partial s}{\partial Q_{ij}^{a}}=-\alpha_{2}^{a}Q_{ij}^{a}.$ From (66), the entropy production, using expressions (1), (3) and (65), assumes the form

$$\sigma^{s}=-\alpha_{1}J^{\left(q\right)}\cdot {\dot{J}}^{\left(q\right)}+J^{\left(q\right)}\cdot \nabla T^{\left(-1\right)}-\beta_{1}^{s}\left[J^{\left(q\right)}\cdot \left(\nabla \cdot Q^{s}\right)+Q^{s}:{\left(\nabla J^{\left(q\right)}\right)}^{s}\right]-\beta_{1}^{\left(a\right)}\nabla \cdot \left(Q^{\left(a\right)}\times J^{\left(q\right)}\right),$$

where we have decomposed $\nabla J^{\left(q\right)}$ in its symmetric part ${\left(\nabla J^{\left(q\right)}\right)}^{s}$ and its antisymmetric part. Using the expression $\nabla \cdot \left(Q^{\left(a\right)}\times J^{\left(q\right)}\right)=J^{\left(q\right)}\cdot \nabla \times Q^{\left(a\right)}-Q^{\left(a\right)}\cdot \nabla \times J^{\left(q\right)}$ we have

$$\sigma^{s}=J^{\left(q\right)}\cdot \left[\nabla T^{\left(-1\right)}-\alpha_{1}{\dot{J}}^{\left(q\right)}-\beta_{1}^{s}\nabla \cdot Q^{s}-\beta_{1}^{\left(a\right)}\nabla \times Q^{\left(a\right)}\right]+Q^{s}:\left[-\alpha_{2}^{s}{\dot{Q}}^{s}-\beta_{1}^{s}{\left(\nabla J^{\left(q\right)}\right)}^{s}\right]+$$

(67) $$Q^{\left(a\right)}\cdot [-\alpha_{2}^{\left(a\right)}{\dot{Q}}^{\left(a\right)}+\beta_{1}^{\left(a\right)}\left(\nabla \times \left(J^{\left(q\right)})\right)\right],$$

We assume the following linear relations among the thermodynamic fluxes $J^{\left(q\right)}$, $Q^{s}$, $Q^{\left(a\right)}$ and the forces $\;[\nabla T^{\left(-1\right)}-\alpha_{1}{\dot{J}}^{\left(q\right)}-\beta_{1}^{s}\nabla \cdot Q^{s}-\beta_{1}^{\left(a\right)}\nabla \times Q^{\left(a\right)}],\;$$\;[-\alpha_{2}^{s}{\dot{Q}}^{s}-\beta_{1}^{s}{\left(\nabla J^{\left(q\right)}\right)}^{s}],\;$ $[-\alpha_{2}^{\left(a\right)}{\dot{Q}}^{\left(a\right)}+\beta_{1}^{\left(a\right)}\left(\nabla \times \left(J^{\left(q\right)})\right)\right]$ as evolution equations for $J^{\left(q\right)}$, $Q^{s}$ and $Q^{\left(a\right)}$

(68) $$J^{\left(q\right)}=\lambda_{1}T^{2}\left[\nabla T^{\left(-1\right)}-\alpha_{1}{\dot{J}}^{\left(q\right)}-\beta_{1}^{s}\nabla \cdot Q^{s}-\beta_{1}^{\left(a\right)}\nabla \times Q^{\left(a\right)}\right],$$

(69) $$Q^{s}=\lambda_{2}^{s}\left[-\alpha_{2}^{s}{\dot{Q}}^{s}-\beta_{1}^{s}{\left(\nabla J^{\left(q\right)}\right)}^{s}\right],\;Q^{\left(a\right)}=\lambda_{2}^{\left(a\right)}[-\alpha_{2}^{\left(a\right)}{\dot{Q}}^{\left(a\right)}+\beta_{1}^{\left(a\right)}\left(\nabla \times \left(J^{\left(q\right)})\right)\right],$$

with $\lambda_{1},\lambda_{2}^{s},\lambda_{2}^{\left(a\right)}$ positive scalar coefficients. If the terms in $\alpha_{2}^{s}$ and $\alpha_{2}^{\left(a\right)}$, proportional to the relaxation terms of $Q^{s}$ and $Q^{\left(a\right)},$ concerning the contributions in ${\dot{Q}}^{s}$ and ${\dot{Q}}^{\left(a\right)},$ may be neglected, one has

(70) $$Q^{s}=-\lambda_{2}^{s}\beta_{1}^{s}{\left(\nabla J^{\left(q\right)}\right)}^{s},\;Q^{\left(a\right)}=\lambda_{2}^{\left(a\right)}\beta_{1}^{\left(a\right)}(\nabla \times \left(J^{\left(q\right)})\right).$$

From (70) and (68) and the expression $\nabla \cdot \left[{\left(\nabla J^{\left(q\right)}\right)}^{s}\right]=\nabla (\nabla \cdot J^{\left(q\right)})+\Delta J^{\left(q\right)}$, one obtains

(71) $$J^{\left(q\right)}=-\lambda_{1}\nabla T-\tau {\dot{J}}^{\left(q\right)}+l_{1}^{2}\left[\nabla \left(\nabla \cdot J^{\left(q\right)}\right)+\Delta J^{\left(q\right)}\right]-l_{2}^{2}\nabla \times \left(\nabla \times J^{\left(q\right)}\right),$$

where $\tau =\lambda_{1}T^{2}\alpha_{1},\;l_{1}^{2}=\frac{1}{2}\lambda_{1}\lambda_{2}^{s}{\left(\beta_{1}^{s}\right)}^{2},\;l_{2}^{2}=\lambda_{1}\lambda_{2}^{\left(a\right)}{\left(\beta_{1}^{\left(a\right)}\right)}^{2}.$ The last term in (71) explicitly describes the relation between ${\dot{J}}^{\left(q\right)}$ and $\nabla \times (\nabla \times J^{\left(q\right)})$.

From the point of view of hydrodynamic analogies, such a development incorporating an antisymmetric part of Q would be similar to the thermodynamic formulation of so-called micropolar fluids (REF), which are fluids of elongated molecules whose rotational degrees of freedom interact with the vortices and give rise to a rotational viscosity, which is not found in fluids of small spherical molecules. In the case of phonon hydrodynamics, such kinds of additional terms could be present in crystals of polar molecules or of elongated molecules, in such a way that the particles could vibrate with respect to their equilibrium position, but could also rotate with respect to such an equilibrium position. In this case, the heat flux would contain contributions coming from the vibrations (the usual phonon contributions) plus contributions related to the rotational energy, which could interact with the phonon vortices of phonon hydrodynamics [107,108,109].

These equations refer to terms contributing to the entropy production. If one makes the additional hypothesis that the reversible part without dissipation has a Hamiltonian structure, as in GENERIC, additional terms may arise in (71), as for instance the Fourier–Crocco term having the form $J^{\left(q\right)}\times \left(\nabla \times J^{\left(q\right)}\right)$ [71], when this term is present and if $l_{1}^{2}$ and $l_{2}^{2}$ are neglected, one obtains

(72) $$\lambda_{1}\nabla T=-J^{\left(q\right)}-J^{\left(q\right)}\times \left(\nabla \times J^{\left(q\right)}\right),$$

according to which, for high $\nabla \times J^{\left(q\right)}$, the vectors $\nabla T,J^{\left(q\right)}$ and $\nabla \times J^{\left(q\right)}$ would be mutually orthogonal.

## 11. Conclusions

In this paper, we have proposed that in some occasions, instead of considering local internal variables obeying non-local evolution equations, one could use non-local internal variables obeying non-local evolution equations. For instance, we have proposed that instead of a local variable $J^{\left(q\right)}$, one could use as a variable $\alpha (J^{\left(q\right)}+R^{2}\Delta J^{\left(q\right)})$, with *R* as the characteristic length of the elementary observation volume. If this observation scale is very small, such an additional term would be negligible, but in the case *R* is sufficiently big, and if within the region having characteristic length *R* the system is still inhomogeneous, $J^{\left(q\right)}$ will have some local variations. Thus, $J^{\left(q\right)}$ would reflect the average value of $J^{\left(q\right)}$ in this volume, whereas the term in $R^{2}\Delta J^{\left(q\right)}$ would describe in some way the dispersion of $J^{\left(q\right)}$ in the volume of size *R*.

As an illustration we have considered a macroscopic model for phonon hydrodynamics, valid when the Knudsen number is comparable to (or bigger than) 1, within the framework of non-equilibrium thermodynamics with internal variables, following the Gyarmati wave theory. A particular motivation was to consider situations where thermal vortices may be of interest. In this case, *R* could be related to the characteristic radius of the thermal vortices, and the corresponding equation generalizes the Guyer–Krumhansl equation, in agreement with some results worked out in [71], where the phonons are treated as a Bose gas within a multiscale thermodynamics.

A general polar vectorial internal variable, having odd parity, was introduced in the thermodynamic state vector, besides the internal energy, without being identified a priori. A very general three-dimensional transport equation for the heat flux was obtained. Therefore, choosing in a suitable way the non-local internal variable, new heat equations were derived that could suggest new thermal phenomena in complex rigid media, such as ferroelectric or/and ferrimagnetic crystals, accounting for the interactions among the polarizations (or magnetizations) of the different molecular species of the crystal and the crystal lattice, and the shell–shell interactions among the polarization (or magnetization) gradients [110,111]. Furthermore, the obtained heat conduction equations can be valid for materials with defects [112]. For instance, in [71], where the phonons are treated as a Bose gas, and vorticity of the heat flux was found, it was supposed the presence of a hydrodynamic force due, for instance, to an insulated obstacle inside a rigid body, that creates a region of lower temperature and then a flow of phonons behind the obstacle (a defect as an example).

In Section 10, we have also discussed the relevant role of the entropy flux in the non-local terms of the evolution equations for the heat transport. Non-local equations for the heat flux are obtained, for instance, assuming that the entropy depends on the internal energy density, the heat flux, and a tensorial internal variable. Since we are interested in situations with thermal vortices, we have proposed to consider that such a tensorial internal variable has a symmetric part and an antisymmetric part, which may be written in terms of a axial vector. A posteriori one may identify the symmetric part of the internal variable as being proportional to the symmetric part of the gradient of the heat flux and the axial vector corresponding to the antisymmetric part as being proportional to the rotational of the heat flux, characterizing the vortices. From the point of view of hydrodynamic analogies, such a development incorporating an antisymmetric part of the internal variable would be similar to the thermodynamic formulation of so-called micropolar fluids, which are fluids of elongated molecules whose rotational degrees of freedom interact with the vortices and give rise to a rotational viscosity.

## Data Availability

The authors agree with the availability of their research data.

## Appendix A

In this Appendix, we give a two-dimensional symmetric representation of the conductivity matrix $\left\{L_{\alpha \beta }\right\}$. This form is useful when the conditions of positive definiteness of the entropy production have to be calculated. Thus, the entropy production (21) can be also written in the symbolic matrix notation

(A1) $$X_{\alpha }L_{\alpha \beta }X_{\beta }\geq 0,$$

where, called $-T^{-1}\frac{\partial T}{\partial x^{i}}=X_{i}^{\left(q\right)}$, $X_{\alpha }$ and $X_{\beta }$ are given by

(A2) $$\left\{X_{\alpha }\right\}=\left\{X_{i}^{\left(q\right)},\;\zeta_{i}\right\},\;\left(i=1,2,3;\;\alpha =1,2,\ldots ,6\right),$$

(A3) $$\left\{X_{\beta }\right\}=\left\{\begin{array}{c} X_{j}^{\left(q\right)}\\ \zeta_{j} \end{array}\right\},\;\left(j=1,2,3;\;\beta =1,2,\ldots ,6\right),$$

and $L_{\alpha \beta }$, in the anisotropic case, is as follows:

(A4) $$\left\{L_{\alpha \beta }\right\}=\left(\begin{array}{cc} \overset{}{\overset{3\times 3}{L_{ij}^{\left(q,q\right)}}} & \overset{3\times 3}{0}\\ \overset{3\times 3}{0} & \overset{}{\overset{3\times 3}{L_{ij}^{\left(1,1\right)}}} \end{array}\right)\;\left(\alpha ,\beta =1,2,\ldots ,6\right),$$

in which $\overset{3\times 3}{0}$ is the symbolic null matrix of dimension 3×3.

Inequalities can be derived for the components of the constitutive tensors, because all the elements of the main diagonal of the matrix $\left\{L_{\alpha \beta }\right\}$ must be non-negative, i.e., $L_{ii}^{\left(q)(q\right)}\geq 0,L_{ii}^{\left(1)(1\right)}\geq 0,\left(i=1,2,3\right)$ and all the major minors of this matrix of even and odd order, must also be non-negative. For example, from the non-negativity of the first two principal minors, we have

(A5) $$L_{11}^{\left(q)(q\right)}\geq 0,\;L_{11}^{\left(q)(q\right)}L_{22}^{\left(q)(q\right)}-{\left(L_{12}^{\left(q)(q\right)}\right)}^{2}\geq 0.$$

In the isotropic case, the entropy production (30) is still written in the symbolic matrix notation (A1), with $X_{\alpha }$ and $X_{\beta }$, given by the expressions (A2) and (A3) and the matrix $L_{\alpha \beta }$ has the following form:

(A6) $$\left\{L_{\alpha \beta }\right\}=\left(\begin{array}{cc} \overset{3\times 3}{L^{\left(q,q\right)}}\delta_{ij} & \overset{3\times 3}{0}\\ \overset{3\times 3}{0} & \overset{3\times 3}{L^{\left(1,1\right)}}\delta_{ij} \end{array}\right)\;\left(\alpha ,\beta =1,2,\ldots ,6\right).$$

Inequalities can be derived for the components of the constitutive tensors, as for instance $L^{\left(q,q\right)}\geq 0,\;L^{\left(1,1\right)}\geq 0.$

## Appendix B

In Section 6, we have assumed that the entropy function s=s(u,ζ) for an isotropic medium has the form (23) $\;s\left(u,\zeta \right)=s^{\left(eq\right)}\left(u\right)-\frac{1}{2}\eta \zeta^{2}.$

Having to be the entropy *s* a concave function, assuming the maximum value at the thermodynamic equilibrium, taking into account that $\frac{\partial s}{\partial u}=\frac{1}{T}=c_{v}\frac{1}{u},\;\frac{\partial s}{\partial \zeta }=-\eta \zeta ,\;u=c_{v}T$, then the Hessian matrix *H* of *s* must be negative-definite, having the form $H=\left(\begin{array}{cc} -c_{v}/u^{2} & 0\\ 0 & -\eta \end{array}\right),$ from which it follows that *H* is negative-definite if the following conditions hold $\;\;-\frac{c_{v}}{u^{2}}<0,\;\frac{c_{v}}{u^{2}}\eta >0,$ i.e., $\;\;c_{v}>0.\;\eta >0.$
